# Identification of Biomolecules Involved in the Adaptation to the Environment of Cold-Loving Microorganisms and Metabolic Pathways for Their Production

**DOI:** 10.3390/biom11081155

**Published:** 2021-08-04

**Authors:** Eva Garcia-Lopez, Paula Alcazar, Cristina Cid

**Affiliations:** Molecular Evolution Department, Centro de Astrobiologia (CSIC-INTA), Torrejón de Ardoz, 28850 Madrid, Spain or garciale@cab.inta-csic.es (E.G.-L.); palc7320@gmail.com (P.A.)

**Keywords:** biomolecules, microorganisms, proteins, metabolites, pigments, antibiotics, antifreeze molecules, lipids, omics, metabolic routes

## Abstract

Cold-loving microorganisms of all three domains of life have unique and special abilities that allow them to live in harsh environments. They have acquired structural and molecular mechanisms of adaptation to the cold that include the production of anti-freeze proteins, carbohydrate-based extracellular polymeric substances and lipids which serve as cryo- and osmoprotectants by maintaining the fluidity of their membranes. They also produce a wide diversity of pigmented molecules to obtain energy, carry out photosynthesis, increase their resistance to stress and provide them with ultraviolet light protection. Recently developed analytical techniques have been applied as high-throughoutput technologies for function discovery and for reconstructing functional networks in psychrophiles. Among them, omics deserve special mention, such as genomics, transcriptomics, proteomics, glycomics, lipidomics and metabolomics. These techniques have allowed the identification of microorganisms and the study of their biogeochemical activities. They have also made it possible to infer their metabolic capacities and identify the biomolecules that are parts of their structures or that they secrete into the environment, which can be useful in various fields of biotechnology. This Review summarizes current knowledge on psychrophiles as sources of biomolecules and the metabolic pathways for their production. New strategies and next-generation approaches are needed to increase the chances of discovering new biomolecules.

## 1. Introduction

**Figure 1:**
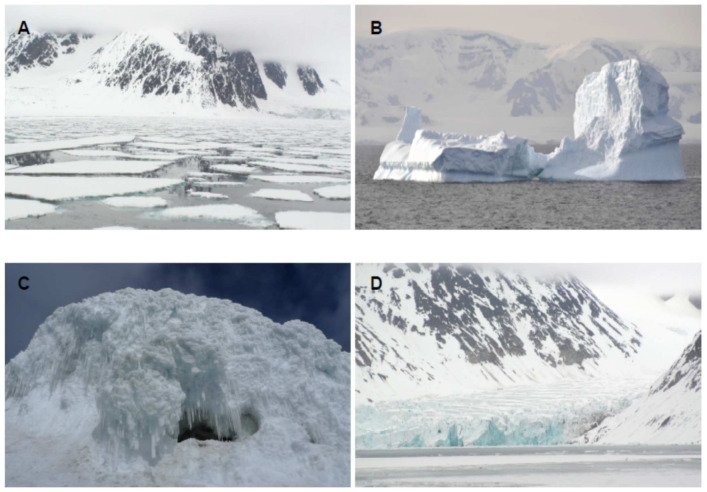
Representative environments inhabited by cold loving microorganisms. (**A**) Sea ice. (**B**) Iceberg. (**C**) Ice cave. (**D**) Glacier.

The identification and characterization of new biomolecules from natural resources is a major challenge for today’s society. Therefore, new strategies and next-generation approaches are needed. Frozen environments constitute an ecological niche inhabited by microorganisms with very remarkable characteristics that produce specific biomolecules. Nowadays, the cryosphere covers about one-fifth of the surface of the Earth [1]. This includes the polar ice sheets, sea ice, ice caves and mountain glaciers (Figure 1). The discovery of cold-tolerant microorganisms in permanently frozen environments has broadened the known range of environmental conditions which support microbial life. Most of the biomass of the cryosphere is made up of microorganisms. There are various classifications for cold-loving microorganisms, but the most used is that of Morita [2], which differentiates between psychrophiles and psychrotolerants or psychrotropes. As this review is more focused on biomolecules than on microorganisms, we will refer to all of them generically as psychrophiles [3].

Microbial activity at cold temperatures is restricted to small amounts of unfrozen water inside the permafrost soil or the ice, and to seawater. Life thrives in these environments with an extraordinary microbial diversity of bacteria, archaea, fungi (in particular yeasts) and microalgae. The freezing process represents an important threat for the survival of life, as growing ice crystals can break cells and disrupt their membranes. Temperatures below 0 °C slow down cellular reaction rates by altering the functionality of the molecular building blocks. However, sometimes cells do not only tolerate this extreme environment, but require it to live. It is well documented that the temperature ranges of microbial habitats play a major role in the selection and adaptation of the resident microorganisms, and hence in microbial diversification [4]. Cold-adapted microorganisms possess molecules (lipids and proteins) in their cellular membranes which provide a flexible interface with the environment for the continued uptake of nutrients and the release of by-products. In this way, they maintain homeostasis and biochemical catalysis at low temperatures.

Cold-loving microorganisms are important because they teach us biochemical fundamentals and structural diversity, and because of their enormous potential as sources of enzymes and other biological materials with applications in biotechnology, molecular biology and pharmacology [5]. Several reviews detail their so diverse uses [6,7,8].

In recent years, a plethora of new techniques, notably omics, have revolutionized the study of environmental microorganisms. Omics include genomics, transcriptomics, proteomics, glycomics, lipidomics and metabolomics. These techniques have made it possible to discover a microscopic frozen world populated by microorganisms of a very diverse nature, capable of adapting to an extreme environment. These techniques have allowed us to know how these organisms live, and what types of adaptations to the environment allow them to survive.

In this Review, we summarize our current knowledge of the biomolecules involved in the adaptation to the environment of cold-loving microorganisms and the metabolic pathways they use to produce these biomolecules. In addition, we will examine the most relevant techniques that are being used today for the study of microorganisms and their bioproducts.

## 2. Biomolecules That Are Protective against the Cold

All cold-loving microorganisms seem to share similar strategies for living in ice, although one of the most important is the synthesis of biomolecules protective against cold. Several biomolecules deserve special mention: antifreeze molecules, pigments, proteins and lipids.

### 2.1. Antifreeze Molecules

Microorganisms in cold environments are able to survive in adverse conditions thanks to the production of cryo- and osmoprotectant molecules such as anti-freeze proteins and carbohydrate-based extracellular polymeric substances (EPS). Most EPS consist of heteropolysaccharides made up of three or four different monosaccharides, usually pentoses, hexoses, amino sugars or uronic acids [9]. The fundamental functions of EPS are the promotion of cell aggregation by modifying their physical properties, and enabling the formation of biofilms within and under ice. All this constitutes an important strategy for the growth and survival of microorganisms in adverse conditions. EPS retain water and act as cryoprotectants (Table 1).

Several ecologic studies have demonstrated that EPS play a main role in polar environments [11,31]. Bacteria such as *Pseudoalteromonas antarctica* NF produce an exopolymeric glycoprotein that provides protection against surfactants [10,32].

Some molecules such as trehalose and exopolysaccharides might play important roles in cryoprotection by preventing protein denaturation and aggregation. High concentrations of EPS modify the physico-chemical environment of bacterial cells and take part in cell adhesion to surfaces and water retention. They also promote nutrient sequestration, retain and protect extracellular enzymes against cold denaturation and act as cryopeotectants [31]. It has been reported that glacial ice itself harbors copious amounts of EPS. The close relationships between sea-ice algal biomass and EPS concentrations, and specific EPS production rates from bacterial and algal cells, suggest that diatoms are the main producers of EPS in these sea-ice habitats [33].

In recent years there has been great interest in the investigation of new polysaccharides from microorganisms. These molecules offer important applications in the industry as gelling agents, viscosifiers, emulsifiers and texture enhancers. In these investigations it has been considered that psychrophiles constitute a valuable source of useful molecules in biotechnological processes. Furthermore, the molecules from psychrophils are being studied to find out how they maintain their stability when subjected to extreme conditions of temperature, radiation or dehydration.

### 2.2. Pigments

Cold adapted microorganisms produce a wide diversity of pigmented molecules to obtain energy [34], to develop photosynthesis [35], to defend against other microorganisms [36], to increase their stress resistance [37] and to act as ultraviolet light protection [38,39].

One example of a cold-adapted microorganism that produces pigments are the psychrotolerant bacterium *Sphingobacterium antarcticus*, which produces zeaxanthin (polar carotenoid), b-cryptoxanthin and b-carotene [18]. Distinct examples are the polar psychrophilic bacteria *Octadecabacter arcticus* and *Octadecabacter antarcticus*, producers of xanthorhodopsin [19], and *Shewanella frigidimarina* isolated from Antarctica, which produces the red cytochrome c3 [20,40] (Table 1). Colored melanized fungi like the ascomycetous and basidiomycetous yeasts, mainly represented by the genera Cladosporium and Aureobasidium, also live in cold environments [21].

In addition to protection against solar radiation, the pigments of psychrophilic microorganisms have another fundamental utility. These pigments increase the absorption of light and solar heat on glacial surfaces, facilitating the fusion of snow and ice. This increases the availability of liquid water and the dissolution of nutrients that can be used by microbial communities [41].

### 2.3. Proteins

#### 2.3.1. Enzymes

Enzymes are the biomolecules of cold loving microorganisms that have been investigated most due to their multiple industrial applications. Therefore, there are very extensive and detailed publications on the subject [42]. Psychrophiles and their enzymes are characterized by high catalytic activity and can operate at temperatures of up to −20 °C. A range of structural features correlate with enzyme cold adaptation. However, there is no structural feature that is present in all cold-adapted enzymes, and there are no structural features that are always correlated with cold adaptation [42]. The cold adaptation of enzymes is achieved by increasing the flexibility of their structure, which can affect the entire protein or be restricted to the areas involved in catalysis [43].

The intimate link between conformational flexibility and protein function carries an important implication for the evolution of protein stability. To work quickly and accurately, proteins cannot become too rigid, at least in those regions of the molecules that are involved in ligand recognition, and that undergo conformational changes during the catalytic cycle. Thus, natural selection prevents proteins from acquiring the highest degree of structural stability possible [44]. For instance, some of the main barriers to protein synthesis at low temperatures include the reduced activity of transcriptional and translational enzymes, reduced protein folding and stabilization of DNA and RNA secondary structures. In psychrophiles, enzymes involved in these processes have adapted to be optimally active at low temperatures.

A variety of enzymes, including proteases, amylases, lipases and cellulases, are used as components of detergents and personal care products. Psychrophilic enzymes have got numerous applications in food, cosmetic and detergent industries; in drug discovery [15]; and as environmental biosensors [45]. These enzymes can catalyze many reactions at low and moderate temperatures (<40 °C) with higher efficiency and fewer unwanted chemical reactions than at higher temperatures, decreasing energy cost and expense [46]. Cold-adapted enzymes are also being used for processing foods, due to their high catalytic activity at low temperatures, which minimizes spoilage and alterations in taste and nutritional values. Other enzymes from psychrophiles that are of great interest enable cost-effective lignocellulose biomass conversion, thereby facilitating the development of economically viable ethanol production from agricultural waste, forestry waste, energy crops and municipal solid waste.

#### 2.3.2. Other Proteins

When bacteria cultures are shifted from their optimal growth temperatures to lower temperatures, cells stop growth immediately and the synthesis of most proteins is repressed. It takes several hours to resume full translation, and during this acclimation phase, the synthesis of some proteins increases. These proteins, termed cold shock proteins (Csps), are encoded by cold shock genes [22]. Csps have been widely studied in model organisms such as *Escherichia coli*. In this bacterium, Csps are nucleic-acid binding proteins that take part in several processes, such as DNA replication, transcription and translation. Among Csps, the CspA family constitutes the main cold shock response, with 10% of the total protein synthesis [47,48]. Furthermore, *E. coli* encode for other Csps such as CspB and CspG, which together with CspA act as RNA chaperones.

All these Csps, firstly studied in model organisms as *E. coli*, have also been found in many other mesophilic and psychrophilic bacteria.

Psychrophiles do also contain antifreeze proteins (AFPs) that can bind to ice crystals through a large complementary surface and create thermal hysteresis, lowering the temperature at which an organism can grow [23]. The full biotechnological potential of AFPs has not been fully exploited. They possess promising applications in cryopreservation, as additives in CO_2_ hydrate slurry production and frozen food preparation [23].

Some Antarctic sea ice bacteria, such as the Gram-negative genus *Colwellia*, produce extracellular substances that change the morphology of growing ice. These active substances have been identified as low molecular weight proteins [25]. The predicted amino acid sequence was similar to predicted sequences of ice-binding proteins found in sea ice diatoms [49] and a species of snow mold. The production of ice-binding proteins by a range of psychrophilic eukaryotic algae may also explain the success of phototrophic eukaryotes in cold habitats [50].

### 2.4. Lipids

The lipid composition governs the physical properties of cell membranes and varies with the thermal habitat of microorganisms. Low temperatures produce a reduction in cell membrane fluidity. Thus, cold-loving microorganisms change the lipidic structure of their membranes to maintain the flexibility of the lipid bilayer. This modified composition has not been found in other microorganisms [43]. It is achieved by altering the order of the lipid chains, or by synthesizing lipids with cis-unsaturated double bonds that induce a link in the acyl chain. Other strategies are the introduction of steric constraints through the incorporation of branched chains or short fatty-acyl chains that prevent contact between adjacent chains [51].

## 3. Metabolic Pathways to Produce Biomolecules

The increasing number of complete genome sequences enables comparative analysis of the general metabolic capacity. It has been suggested that there are some enzymatic conversions for which genes are not yet known, and for other metabolic pathways some expected genes have not been found [52].

Recently developed proteomic techniques for determining gene function along with differential expression detection and analysis have been applied as high-throughput technologies for function discovery and for reconstructing functional networks in psychrophiles.

New strategies and next-generation approaches are needed to increase the chances of discovering biomolecules. Some examples of the production of biomolecules by psychrophilic microorganisms are described below.

### 3.1. Production of Antimicrobial Compounds

Actinobacteria and fungi are the sources of approximately two-thirds of the antimicrobial agents currently used in human medicine [53]. Antimicrobial compounds include a wide variety of natural products and secondary metabolites that are not required for survival under laboratory conditions but provide some advantages in the environment. Antarctic bacteria have been extensively studied as a source of new antibiotics. Among them, Actinobacteria (*Streptomyces fildesensis* and *Microbacterium* sp.), Proteobacteria (*Sphingomonas alpina*, *Stenotrophomonas maltophilia* and *Massilia* sp.) and Firmicutes (*Bacillus subtilis*) are capable of producing or increasing the production of secondary metabolites under culture and elicitation conditions [54]. Several strategies have been used in the production of new antibiotics. In addition to the aforementioned cultures, using special conditions that promote the synthesis of certain molecules, new methods are being used:

#### 3.1.1. Genome Mining

The genomics of microorganisms have generated a large number of genomic sequences. Their analysis has revealed the existence of biosynthetic gene clusters (BGCs), encoding proteins predicted to be involved in the biosynthesis of natural products, but in many cases, the metabolic products of these genes are not known. Thus, genome mining tries to establish a relationship between the identification of unknown natural products and the possible genomes involved in their production. The bioinformatics tools most used in genome mining are the approaches based on genetic sequences, ecology, mode of action and the relationship between function and genomes [53].

#### 3.1.2. CRISPR-Cas

Another strategy with which to design antimicrobial compounds with predetermined activities is the use of CRISPR (clustered regularly interspaced short palindromic repeats)/Cas (CRISPR-associated protein). Bacteria and archaea have evolved many diverse strategies to defend themselves against phage predation. Among them, CRISPR–Cas systems target and inactivate specific nucleic acid sequences by cleavage. Specifically, the type II CRISPR-Cas9 system has been widely adapted for genome editing and used in many biotechnological applications [55].

### 3.2. Production of Pigments

#### 3.2.1. Carotenoids

**Figure 2:**
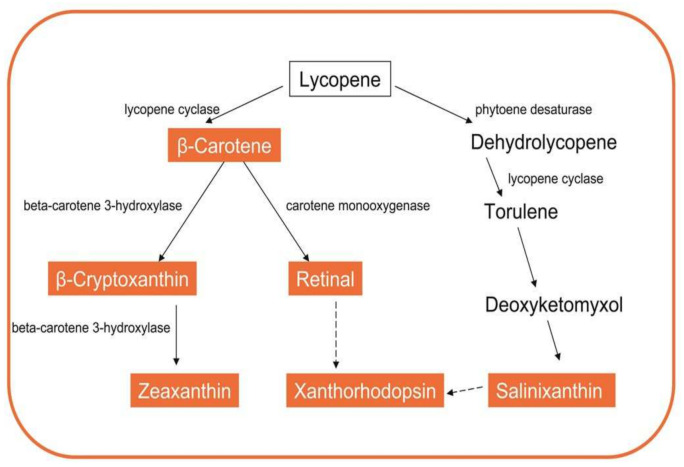
Metabolic pathways for the synthesis of representative biomolecules carotenoids.

In the polar psychrophilic bacteria *Octadecabacter*, the synthesis of carotenes such as zeaxanthin and beta-cryptoxanthin from beta-carotene is catalyzed by the enzyme beta-carotene 3-hydroxylase [18]. This enzyme requires ferredoxin and Fe (II). Other Antarctic actinobacteria *Marisediminicola antarctica* synthesize sreddish-orange pigments at low temperatures, sharing the characteristics of carotenoids. These chromophores were identified as salinixanthin, retinal and xanthorhodopsin. Proposed pathways for the biosynthesis of these pigments are represented in Figure 2. The common precursor to all of them is lycopene, which can be cyclized by the enzyme lycopene cyclase, giving rise to the synthesis of carotene or torulene. From these compounds, and following a multi-stage process, the aforementioned pigments are obtained.

#### 3.2.2. Melanins

**Figure 3:**
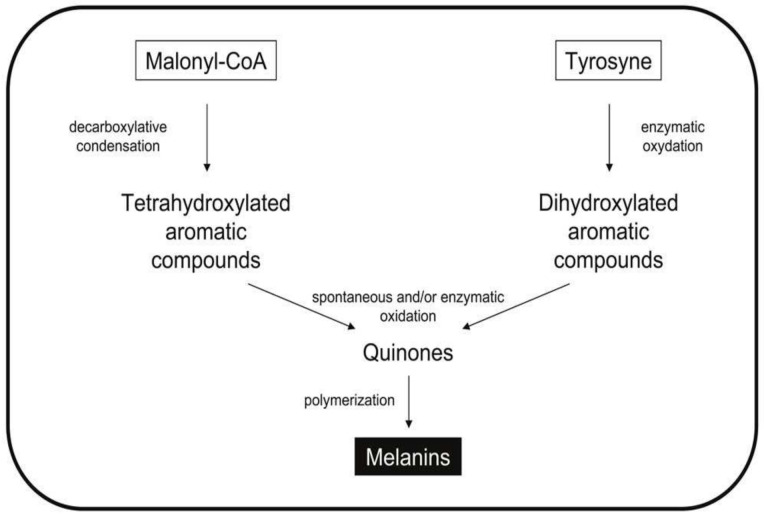
Metabolic pathways for the synthesis of melanins.

Melanins are a group of black pigments widely distributed in animals, plants, fungi and bacteria [56] (Figure 3). They are secondary metabolites made up of complex heterogeneous polymers of phenolic and/or indolic monomers [57]. In nature, these molecules protect microorganisms from environmental stress, oxidative stress, stress caused by ultraviolet radiation and toxic heavy metals. Melanins are not a single compound with a specific structure, but a set of black pigments derived from aromatic amino acids such as tyrosine. These biopolymers are classified into different types according to their chemical natures, their synthesis pathways and the particular enzymes involved in their production (Figure 3).

### 3.3. Production of Hopanoids

**Figure 4:**
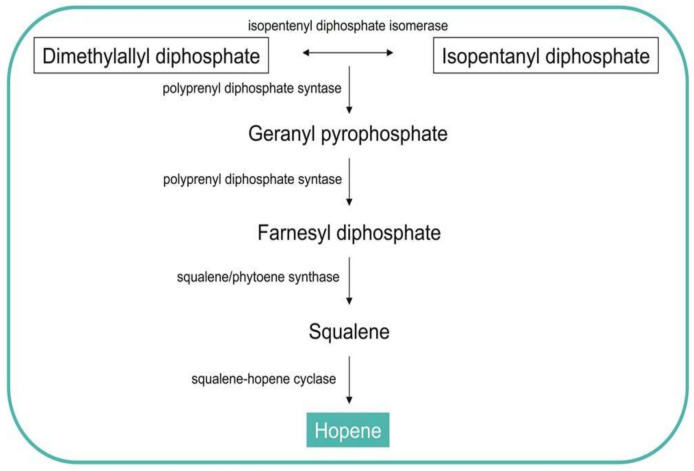
Metabolic pathways for the synthesis of Hopenes.

Hopanoids comprise a class of pentacyclic triterpenoid lipids found in a wide range of Gram-positive and Gram-negative bacteria [58]. These molecules are involved in regulating membrane fluidity and stability. Hopene is cyclized from squalene, which is, in turn, synthesized in several steps from the precursors isopentenyl diphosphate (IPP) and its allylic isomer dimethylallyl diphosphate (DMAPP) (Figure 4). In *Streptomyces* the genes of the enzymes that can catalyze the reactions of the intermediate metabolites have been sequenced to better understand the role of the hopanoids as membrane reinforcers [58].

## 4. Cultures in Different Media and Environmental Conditions

To study a microorganism, it is necessary to explore its physiology, energy metabolism, preferred nutrient sources, responses to environmental stressors and strategies for growth and survival. Therefore, cultivation cannot be replaced by any other approach. The full diversity of the microbial world needs to be explored by cultivating new microorganisms, and by studying them in pure or enriched cultures [59].

Although it was traditionally reported that most of the microorganisms found by DNA sequencing were not cultivable, some researchers have demonstrated that culturing many samples in several culture media and subsequent functional genomic and metaproteomic analyses are possible [60,61]. This method, known as culturomics, can allow both the identification of proteins involved in microorganism metabolism, and the identification of microorganisms that take part in biogeochemical cycles [62,63,64]. In cell cultures, specific environmental conditions (temperature, lighting, oxygen concentration, nutrients, agitation, etc.) can be used to elicit the production of certain metabolites [54]. Table 2 summarizes several culture media used in different reports to perform culturomics and to elicit the production of certain metabolites.

## 5. Omics in Cold-Loving Microorganisms

**Figure 5:**
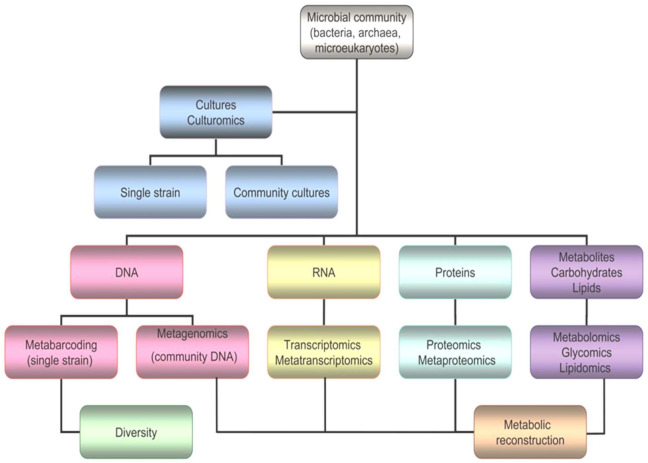
Workflow for omics, including genomics, transcriptomics, proteomics, glycomics, lipidomics and metabolomics.

Nevertheless, it is true that the vast majority of polar microorganisms cannot be cultured or isolated, and new strategies and next generation approaches are needed to increase the chances of drug discovery (Figure 5). Omics comprise several and very different techniques, such as genomics, transcriptomics, proteomics, glycomics, lipidomics and metabolomics [67].

### 5.1. Genomics

Genomics aims at the characterization, sequencing and analysis of genomes. In environmental microbiology, this discipline has revolutionized the understanding of microbiomes, and in frozen environments, it has meant unprecedented advances. Knowledge of the microbial genome is essential for the development of other omics, such as proteomics, which predicts the amino acid sequences from the genetic sequences of the organisms to be studied. To identify the proteome of a microorganism, it is essential to know its genome first. The best-known proteomes are those from “model species” whose genomes were completely sequenced years ago, and their comprehensive study by other omics also occurred very fast. However, other less genetically studied environmental microorganisms have encountered the stumbling block of a lack of genetic knowledge.

Nowadays, genomics comprises two large groups of techniques: metabarcoding and metagenomics.

#### 5.1.1. Metabarcoding

Metabarcoding targets a specific DNA region in the community genome, and it is used to study microbial diversity. The DNA regions identified are usually 16S rRNA for bacteria and 18S rRNA for microeukaryotes. Primers of the regions V3 and V4 of the 16S rRNA gene, or of the V4 and V5 regions of the 18S rRNA gene are used for the amplification of these DNA regions. For example, metabarcoding has been recently used to overview the diversity of bacterial and microeukaryotic communities in several Arctic and Antarctic glaciers [45,68].

Metabolic reconstruction can be theoretically deduced from taxonomy using specific computer tools such as PICRUSt [69], PanFP [70], Piphillin [71] or Tax4fun [72]. These programs allow inferences about the functional profile of a microbial community based on a marker gene survey, along with one or more sample. They constitute forms of automated searching, in which the functional genes present in the metagenome are deduced from the microorganisms identified by metabarcoding and their relative abundances. An application of this methodology was the metabolic inference, i.e., the systematic prediction, of metabolism from phylogeny in the microbial community from the coastal west Antarctic Peninsula [73].

#### 5.1.2. Metagenomics

Metagenomics consists of the sequencing of the total metagenome in the microbial community, which provides far more detailed information than metabarcoding. The knowledge of the total genome of the microorganisms allows deducing the metabolic potential and the biogeochemical cycles of the community with far greater precision.

The number of available metagenomic analyses from cold environments is rapidly increasing. An example of the application of this technique is a study of the marine microbiota in the Arctic and Antarctic seas, which has also made it possible to reconstruct several metabolic pathways [74]. In that report, the comparison of the Arctic and Antarctic microbiomes showed that in the Arctic microorganisms have a greater number of genes related to resistance to antibiotics, perhaps as a consequence of their exposure to environmental contamination, including human activity. In contrast, in Antarctica, microorganisms have a greater capacity to repair DNA, due to their intense exposure to environmental changes, such as ultraviolet radiation.

In all this research on environmental genomics, the information of the identified and sequenced microorganisms must be deposited in public databases such as SRA from NCBI, the Greengenes 16S rRNA database [75] or the Silva ribosomal RNA sequence datasets [76]. These data are essential to deducing what their cellular machinery, their metabolites and their metabolic pathways are like.

### 5.2. Transcriptomics and Metatranscriptomics

As we have described, genomics applied to DNA samples from cold environments informs us about the existence and diversity of the microorganisms that inhabit them. These techniques can also inform us about the theoretical metabolic potential of microbial communities, but it is not possible to know their capacities for survival and proliferation, nor the molecular mechanisms of adaptation that allow microorganisms to live in such hostile environments. To learn about their physiology and metabolisms, it is necessary to know which RNAs are translated in cells, giving rise to proteins.

Transcriptomic and metatranscriptomic analyses retrieve knowledge about genes expressed under certain conditions. These studies have yielded important insights into the mechanisms and metabolisms that allow psychrophiles to remain active in cold environments. Many of these studies about bacteria, archaea, algae, ciliates and diatoms from frozen environments have been reviewed in [77]. These results show numerous changes in the transcriptional profiles at cold temperatures and the regulation of the genes involved in cellular processes such as metabolism, cell wall biosynthesis, cell membrane composition and stress responses.

Besides standard RNA, there are antisense transcripts that contain information regarding other functions. These small non-coding RNAs are mainly involved in regulatory roles, controlling the expression of various genes in a cell under different conditions, including specific stress conditions and environmental or morphological changes. For example, the role of small non-coding RNA has been studied for the control of gene expression in the model organism *Streptomycetes* required to efficiently regulate its complex life cycle [78].

### 5.3. Proteomics and Metaproteomics

Proteomics studies the set of proteins expressed by a cell (proteomics) or set of cells (metaproteomics) under certain conditions, giving rise to high-throughput data. They have been widely used techniques for the study of psychrophilic microbial communities. The proteins to be studied can firstly be separated by chromatographic or electrophoretic techniques and subjected to analysis by mass spectrometry.

Matrix-assisted laser desorption ionization-time of flight mass spectrometry (MALDI-TOF/MS) has emerged as a powerful approach suitable for analyzing not only proteins, but many other macromolecules, such as polysaccharides, lipids and nucleic acids. The ability to precisely determine molecular weight by this technique and to search databases for mass matches has made high-throughput identification possible in many microbial samples [64].

Among microbial proteomes, these techniques have been employed to study biomolecules such as the aforementioned cytochromes, Csps, AFPs or enzymes. On the other hand, proteins constitute the molecular machines that function within cells. The study of molecular machinery elucidates the mechanism of action of biomolecules in cells, or whether molecules induce drug resistance responses. Proteins undergo different post-translational modifications to perform their functions, which modify the detected masses, and in this way they can be detected. Moreover, proteins interact with other proteins, forming protein complexes. These complexes can also be studied by these techniques [66].

### 5.4. Glycomics and Lipidomics

Glycomics and Lipidomics involve the study of carbohydrate (glycome) and lipid (lipidome) profiles, respectively. As mentioned above, many of the molecules that allow the survival of microorganisms at low temperatures are carbohydrates and lipids. Just as nucleic acids and proteins are polymers formed by repeating monomers and can be studied with standardized methodology and found in well-documented databases, carbohydrates and lipids have such disparate molecular structures that their study is very complex.

Glycomics focuses on the characterization and quantification of carbohydrates and their conjugates, including proteoglycans, aminosugars, sulfoglycans and glycolipids. The two major carbohydrate databases of glycomes are the Bacterial Carbohydrate Structure Data Base (BCSDB) and Glycosciences.de (GS) [79].

Lipidomics studies a vast diversity of molecules with very different molecular structures. Even so, mapping the lipid profiles of microorganisms is crucial to studying their metabolic pathways and to finding active biomolecules with biotechnological potential [15]. Some Antarctic bacteria living in sea ice—for instance, *Shewanella* and *Colwellia*—produce polyunsaturated fatty acids, such as eicosapentaenoic acid and docosahexaenoic acid, as components of their membrane phospholipids. These polyunsaturated fatty acids are used in the pharmaceutical industry for their favorable effects on pathologies as diverse as atherosclerosis, diabetes and high blood pressure [15].

### 5.5. Metabolomics

The microbial metabolome is the set of low molecular weight molecules (e.g., metabolic intermediates, hormones, signal molecules and secondary metabolites) contained or excreted from a certain microorganism in a particular time, or under specific environmental conditions. Its study is very helpful because it provides information on the physiological state of a microorganism.

The most widespread techniques for the study of the metabolome are mass spectrometry after separation of the components by various techniques, giving rise to gas chromatography-mass spectrometry (GC-MS), liquid chromatograph-mass spectrometry (LC-MS) and capillary electrophoresis-mass spectrometry (CE-MS). The de novo elucidation of the molecular structure can be done by nuclear magnetic resonance (NMR).

## 6. Conclusions and Perspectives

The microbial communities that populate the cryosphere represent an inexhaustible source of biomolecules with great potential to be used as active principles, as raw materials in the industry, and as useful molecules in “cold biotechnology.” These microorganisms, adapted to frozen worlds, have to cope with a hostile environment that forces them to develop specific adaptation mechanisms and to produce very specific molecules. Additionally, they are parts of the biogeochemical cycles of the ecosystems that they occupy and interact with many other living beings. At present, climate change is seriously threatening their environments, and many of the ecological niches they occupy will be modified or will disappear completely in the future. These microorganisms have also great appeal as biosensors of climate change [45]. Microbial populations, and some indicator species, are sentinels for environmental degradation and alteration in the cycling of globally important elements. Finally, it should be noted that biomolecules from cold-loving microorganisms are of great interest in many other applications, such as astrobiology and space exploration [80].

It is necessary to explore their potential as a source of biomolecules and to promote their study by taking advantage of the large number of opportunities offered by technologies such as omics and bioinformatics. To advance in the discovery of biomolecules from environmental microorganisms, it is necessary to completely sequence their genomes and deposit their sequences in public databases. This information is essential for the development of other techniques such as proteomics. The perspectives include a breakthrough from computer sciences to integrate all information gathered from omics, and to add data that can be theoretically deduced from metagenomic information or from bibliographic references and databases.

## Figures and Tables

**Table 1:** Biomolecule-producing microorganisms.

Microorganism		Biomolecules	References
*Pseudoalteromonas antarctica*	EPS	Glycoprotein	[10]
*Pseudoalteromonas* sp.		Sulfated heteropolysaccharide	[11]
*Pseudoalteromonas* sp.		Linear arrangement of α-(1–6) linkage of glucose with a high degree of acetylation	[12,13]
*Psychrobacter arcticus*		Peptidoglycan	[14]
*Psychrobacter frigidicola*		Peptidoglycan	[15]
*Colwellia psychrerythraea*		N-acetyl quinovosamine and galacturonic acid	[16,17]
*Sphingobacterium antarcticus*	Pigments	Zeaxanthin, b-cryptoxanthin, b-carotene	[18]
*Octadecabacter arcticus*		Xanthorhodopsin	[19]
*Octadecabacter antarcticus*		Xanthorhodopsin	[19]
*Shewanella frigidimarina*		Cytochrome c3	[20]
*Cladosporium* (fungi)		Melanin	[21]
*Aureobasidium* (fungi)		Melanin	[21]
*Escherichia coli*	Proteins	Csps	[22]
*Pseudomonas*, *Arthrobacter*		AFP	[23]
*Chaetoceros neogracile* (diatoms)		AFP	[24]
*Chloromonas brevispina* (alga)		Ice-binding proteins	[25]
*Fragilariopsis cylindrus* (diatoms)		Ice-binding proteins	[26]
*Colwellia*		Ice-binding proteins	[27]
*Arthrobacter*		Enzymes (chitobiase)	[28]
*Synechocystis*	Lipids	Diunsaturated fatty acids	[29]
Antarctic bacterium, strain JS6		Polyunsaturated fatty acids	[30]

**Table 2:** Composition of culture media.

Name	Composition (g/L)	Reference
M1	2 g peptone, 4 g yeast extract, 10 g starch	[54]
ISP2	4 g yeast extract, 10 g malt extract, 4 g glucose	
M2	40 g mannitol, 40 g maltose, 10 g yeast extract, 2 g K_2_HPO_4_, 0.5 g MgSO_4_.7H_2_O, 0.01 g FeSO_4_.7H_2_O	
SCA	10 g starch, 0.3 g casein, 2 g KNO_3_, 2 g NaCl, 2 g K_2_HPO_4_, 0.5 g MgSO_4_.7H_2_O, 0.02 g CaCO_3_, 0.01 g FeSO_4_.7H_2_O	
IMA	4 g yeast extract, 10 g malt extract, 4 g glucose, mannitol	
SDB	p g peptidic digest of animal tissue, 5 g casein pancreatin disgest, 40 g dextrose	
CCA	30 g glicerol, 2 g peptone, 1 g K_2_HPO_4_, 1 g NaCl, 0.5 g MgSO_4_.7H_2_O, 5 mL trace element solution	
YEME	3 g yeast extract, 5 g peptone, 3 g malt extract, 10 g glucose, 170 g sucrose	
GYA	4 g Yeast extract, 10 g malt extract, 4 g glucose, 2 g CaCO_3_, 20 g starch	
YES	150 g sucrose, 20 g yeast extract, 0.5 g MgSO_4_.7H_2_O, 0.01 g ZnSO_4_.7H_2_O, 0.005 g CuSO_4_.5H_2_O	
ISP4	10 g starch, 2 g CaCO_3_, 2 g (NH_4_)_2_SO_4_, 1 g K_2_HPO_4_, 1 g MgSO_4_.7H_2_O, 1 g NaCl, 1 mg FeSO_4_.7H_2_O, 1 mg MnCl_2_.7H_2_O, 1 mg ZnSO_4_.7H_2_O	
R2YE	103 g sucrose, 0.25 g K_2_SO_4_, 0.12 g MgCl_2_.6H_2_O, 10 g Glucose, 0.1 g casaminoacids, 5 mL yeast extract (10%), 1 mL KH_2_PO_4_ (0.5%), 8 mL CaCl_2_.2H_2_O (3.68%), 1.5 mL L-proline (20%), 10 mL TES buffer (5.73%), 0.2 mL trace element solution, 0.5 mL NaOH (1N)	[58]
T2	5 g peptone, 0.15 g ferric ammonium citrate, 0.2 g MgSO_4_.7H_2_O, 0.05 g CaCl_2_, 0.05 g MnSO_4_.H_2_O, 0.01 g FeCl_3_.6H_2_O	[64,65]
T3	1 g glucose, 1 g peptone, 0.5 g yeast extract, 0.2 g MgSO_4_.7H2O, 0.05 g MnSO_4_.4H_2_O	
T4	1 g glucose, 0.5 g casamino acids, 0.5 g yeast extract, 1 g KH_2_PO_4_, 0.5 g CaCl_2_.2H_2_O, 0.5 g MnCl_2_.4H_2_O	
T6	R2A	
T7	Marine broth 2216	[66]
T8	Trypticase soy broth	
